# Effects of Coral Reef Benthic Primary Producers on Dissolved Organic Carbon and Microbial Activity

**DOI:** 10.1371/journal.pone.0027973

**Published:** 2011-11-18

**Authors:** Andreas F. Haas, Craig E. Nelson, Linda Wegley Kelly, Craig A. Carlson, Forest Rohwer, James J. Leichter, Alex Wyatt, Jennifer E. Smith

**Affiliations:** 1 Scripps Institution of Oceanography, University of California San Diego, San Diego, California, United States of America; 2 Marine Science Institute, University of California Santa Barbara, Santa Barbara, California, United States of America; 3 Department of Biology, San Diego State University, San Diego, California, United States of America; 4 Department of Ecology, Evolution and Marine Biology, University of California Santa Barbara, Santa Barbara, California, United States of America; Swansea University, United Kingdom

## Abstract

Benthic primary producers in marine ecosystems may significantly alter biogeochemical cycling and microbial processes in their surrounding environment. To examine these interactions, we studied dissolved organic matter release by dominant benthic taxa and subsequent microbial remineralization in the lagoonal reefs of Moorea, French Polynesia. Rates of photosynthesis, respiration, and dissolved organic carbon (DOC) release were assessed for several common benthic reef organisms from the backreef habitat. We assessed microbial community response to dissolved exudates of each benthic producer by measuring bacterioplankton growth, respiration, and DOC drawdown in two-day dark dilution culture incubations. Experiments were conducted for six benthic producers: three species of macroalgae (each representing a different algal phylum: *Turbinaria ornata* – Ochrophyta; *Amansia rhodantha* – Rhodophyta; *Halimeda opuntia* – Chlorophyta), a mixed assemblage of turf algae, a species of crustose coralline algae (*Hydrolithon reinboldii*) and a dominant hermatypic coral (*Porites lobata*). Our results show that all five types of algae, but not the coral, exuded significant amounts of labile DOC into their surrounding environment. In general, primary producers with the highest rates of photosynthesis released the most DOC and yielded the greatest bacterioplankton growth; turf algae produced nearly twice as much DOC per unit surface area than the other benthic producers (14.0±2.8 µmol h^−1^ dm^−2^), stimulating rapid bacterioplankton growth (0.044±0.002 log10 cells h^−1^) and concomitant oxygen drawdown (0.16±0.05 µmol L^−1^ h^−1^ dm^−2^). Our results demonstrate that benthic reef algae can release a significant fraction of their photosynthetically-fixed carbon as DOC, these release rates vary by species, and this DOC is available to and consumed by reef associated microbes. These data provide compelling evidence that benthic primary producers differentially influence reef microbial dynamics and biogeochemical parameters (i.e., DOC and oxygen availability, bacterial abundance and metabolism) in coral reef communities.

## Introduction

Tropical coral reef ecosystems often support enormous biological diversity [1], including structurally and functionally complex benthic communities [2]. As a result numerous interactions may arise among species [1] and their surrounding environments, making these ecosystems particularly susceptible to changes in environmental parameters [3], [4], [5]. Although ambient oceanographic conditions play a fundamental role in the structure and function of a given reef community, benthic primary producers can significantly alter key biogeochemical parameters (e.g. oxygen and organic carbon concentrations) in their surroundings via basic physiological processes [6], [7], [8].

Dominant primary producers from a broad array of ecosystems are known to release variable quantities of “photosynthate” as dissolved organic carbon (DOC) into their respective surroundings, including terrestrial plants [9], [10], [11], aquatic plants [12], [13], [14] **,** freshwater algae [15], [16], marine algae [17], [18], [19] and hermatypic corals [20], [21], [22]. In coral reef ecosystems, this primary producer-derived DOC is known to affect activity and growth of microbial communities, which in turn play an important role in the remineralization of organic to inorganic constituents [23] or transfer of energy to higher trophic levels [5], [8], [24]–[27]. Modeling studies indicate that a majority of reef primary production enters the food web through microbial consumption processes [28], [29], and the release of extracellular DOC by benthic primary producers may thus play a key role in fueling the reef food web [30]. Moreover, the source, quality, and quantity of this bioavailable organic material may fundamentally regulate variation in overall ecosystem functioning [31], [32].

Among various other ecosystem implications of benthic primary producer derived DOC, specific attention has been given to the role that algal exudates may have on interactions between coral and algae [33]–[37]. Several recent studies have shown that benthic reef algae can directly or indirectly influence coral health and abundance. Direct interactions such as abrasion [34], disease transmission [38]–[41] and allelopathic effects caused by lipid soluble compounds [34], [42] occur between numerous species of reef algae and corals. Indirect interactions have also been suspected to cause coral damage, yet much less is known about the potential mechanisms involved in these interactions. The primary hypothesis relating to indirect coral-algal interactions is that algae may release excess photosynthate into the water column in the form of DOC, which may serve as a food resource for microbes which in turn may increase in abundance on adjacent corals. The increased heterotrophic microbial production can lead to hypoxia and coral death [43], [44], [45]. A few studies have specifically quantified [46], [47] and qualified [48] DOC release rates from a select number of benthic reef algae. However, recent data on algal organic matter release, especially in coral reef ecosystems, are still rare and difficult to compare to earlier studies [e.g. 18,49] due to different incubation and analytical methods as well as different reference parameters.

Given that some benthic reef algae are known to release DOC into the surrounding water, very little is known about how this DOC contributes to reef metabolism and microbial processes. If labile carbon is a limiting resource for microbial communities, DOC released by benthic primary producers may have significant indirect effects on coral health, including changing microbial community structure, perhaps favoring coral pathogens [50], [51], and/or increasing microbial respiration causing subsequent localized hypoxia [52], [53], [54]. Dissolved oxygen (DO) produced by benthic photosynthesizers during daylight hours is rapidly used by the reef community at night as a result of eukaryotic respiration and microbial oxidation of organic material [55]. Temporal and spatial patterns of hypoxia in coral reef environments have been described in several studies, with DO concentrations reaching as low as 2.1 mg oxygen L^−1^ [56] and measurements on interstices of single organisms have revealed concentrations of less than 0.7 mg oxygen L^−1^ [57], [58]. Additional elevated microbial oxygen consumption may further decrease DO concentrations [59] and ultimately lead to hypoxia and coral death [23], [53], [60], [61]. While components of this algal-mediated microbially-induced coral mortality have been anecdotally shown in lab experiments [45] more data are needed to identify the specific mechanisms involved in these interactions. Little is known about DOC exudation in tropical reef algae and the ability of marine microbial communities to utilize this algal-released DOC. Even less is known about how algal-derived DOC may affect overall reef metabolism [47], [54] and what effects this may have on local hypoxia in coral reef environments. Overall, the effects of benthic primary production on the surrounding water column may have considerable influence on ecosystem functioning, and understanding these effects is crucial for the design and implementation of effective management actions aimed at strengthening resilience of coral reef ecosystems.

The goals of the present study were to examine how benthic primary producers directly affect key aspects of seawater chemistry (DO and DOC) and to subsequently examine the impacts of released DOC on ambient reef bacterioplankton growth and respiration. A series of complementary experiments were conducted at the Gump research station in Moorea, French Polynesia, to examine differences in direct and indirect (microbially-mediated) alterations in water chemistry mediated by six common benthic primary producers. Samples of different macroalgae, turf algae, crustose coralline algae (CCA), and coral were subjected to a series of replicate incubations in order to assess: 1) DOC release rates among taxa, 2) connections between photosynthetic performance and DOC release rates and, 3) the growth and respiratory response of the ambient microbial community to primary producer-derived DOC.

## Materials and Methods

### Study site

**Figure 1:**
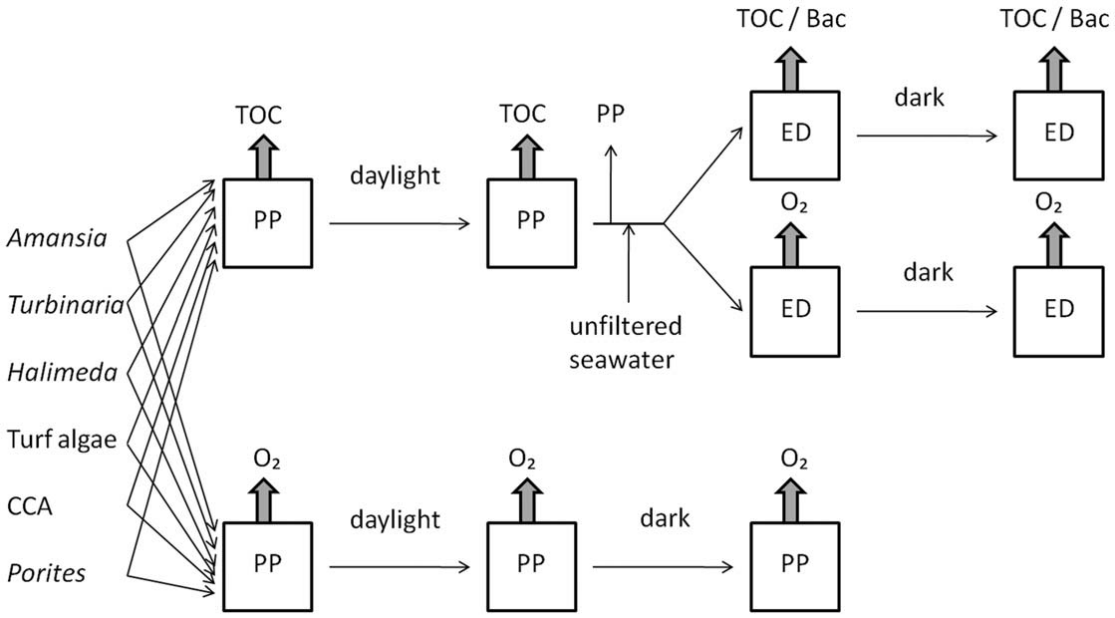
Experimental setup. Primary producers were incubated in filtered backreef seawater in replicates of a minimum of five for each species. Following the 8.5 h daylight DOC release incubations specimen were removed and reference parameters were determined and unfiltered backreef seawater was amended to add a representative microbial community for subsequent dark incubations (48 h) of the exudates. Thick arrows indicate measurements of respective parameters. Abbreviations: PP = primary producer, ED = exudate dilution, TOC = total organic carbon, Bac = bacterial cell abundance.

This study was conducted from 5 to 19 September 2010 at the Richard B. Gump South Pacific Research Station located on the north shore of the island of Moorea, French Polynesia (17°30′S:149°50′W). All research was performed under annual research permits (unnumbered) issued by the French Polynesian Ministry of Research to the Moorea Coral Reef Long Term Ecological Research program (MCR-LTER). Moorea is a high volcanic island roughly 1.5 to 2 million years old [62] encircled by a barrier reef approximately 500 m to 1 km off the coast. The island and its reefs are the site of the multiple long-term ecological research programs, including the MCR-LTER (http://mcr.lternet.edu). The reef ecosystem comprises an outer reef slope, a backreef platform with water depths<3 m, a fringing reef bordering the island, and a large adjacent embayment (Paopao Bay or Cooks Bay). Previous research on bacterioplankton and DOC in the reef system have demonstrated consistent depletion of DOC within the reef relative to offshore waters and shown that backreef bacterial communities are distinct from those outside of the reef crest and the open ocean [63]. The benthic community on the backreef platform, the main area of investigation, is composed of approximately 68.6±4.9% fleshy turf, macroalgae and cyanobacteria, 22.7±4.4% hermatypic coral, 8.1±2.0% sand and 0.5±0.3% other benthic coverage (http://mcr.lternet.edu/data). Six different species of dominant primary producers, together accounting for 78.1±3.7% of all benthic macro organisms in the backreef study area (http://mcr.lternet.edu/data), were selected for subsequent incubation experiments (Figure 1).

### Sample Collection

Six benthic producers including three dominant macroalgal genera (each representing a different algal phylum: *Turbinaria ornata* – Phaeophyta; *Amansia rhodantha* – Rhodophyta; *Halimeda opuntia* – Chlorophyta), a typical mixed consortium of turf algae found in the backreef habitat, a species of CCA (*Hydrolithon reinboldii*) that commonly grows as a rhodolith, and a species of hermatypic coral (*Porites lobata*), were collected from water depths of 1.0–1.5 m at back- and fringing-reef locations. Specimens were collected in replicates of at least 20 individuals or as unattached whole colonies and were transferred in coolers to cultivation tanks within 1 hour using watertight Ziplock® bags. Average surface area of specimens was 2.9±0.4 dm^2^. Specimens infested with epibionts or endolithic boring organisms were excluded to avoid potential confounding of experimental results. No specific permits were required for the described field studies other than the aforementioned general research permits. Studies were not conducted on privately owned or protected property and did not include endangered species.

### Primary producer DOC release

Each benthic species was incubated in an independent experiment using the method of Herndl and Velimirov [64] with minor modifications. Briefly, sulphuric acid-cleaned and seawater-leached polypropylene beakers were filled with exactly 800 mL of ambient seawater, freshly (<15 h) collected from the same sites as the biological samples; 10 beakers contained the selected specimens and 3 served as seawater controls. All incubation waters were filter sterilized (0.2 µm polyethersulfone filter, Millipore SUPOR-200, pre-flushed with 1 L deionized water) to minimize simultaneous DOC consumption by the heterotrophic planktonic microbial community. All beakers were then placed in a flowing seawater bath in a water table with shaded natural light to simulate the conditions in the natural backreef habitat (1.0–1.5 m water depth; mid-day photosynthetically active radiation (PAR) ∼600 µmol quanta m^-2^ s^-1^). To compare to *in situ* conditions, light intensity (lux) and water temperature (°C) were recorded in the cultivation tanks during the incubation experiments and simultaneously in parallel with *in situ* measurements at the natural back-reef sampling location using light and temperature loggers (Figure S1; Onset HOBO® Pendant UA-002-64). These incubations were carried out during daylight hours (09:00 to 17:00 h).

After placing each specimen into randomized beakers, water (60 ml) was sampled from each beaker with a sample-rinsed plungerless HDPE syringe and filtered through a precombusted GF/F filter (Whatman, 0.7 µm nominal particle retention) into precombusted glass vials to determine initial DOC concentrations. Primary producers along with seawater controls were incubated in natural light for 8 h, DOC samples were again collected as described above to determine DOC release rates from all organisms. Specimens were then removed from the beakers using acid-washed forceps and the remaining incubation water was processed for dark dilution remineralization culture incubations (detailed below). Subsequently, wet and dry weights (for *Amansia*, *Turbinaria*, *Halimeda*, turf), surface area (for turf, coral, CCA) and volume (all) of all specimen were determined (detailed below). Specimen volume was always <5% of incubation waters.

### Effects of primary producer physiology on DO

Parallel experiments to the DOC release rates described above were run in airtight chambers to determine the effects of primary producer physiology on DO in the surrounding waters. The two parallel experiments allowed for reliable measurements of DOC release and algal physiology without the potential for contamination of DOC measurements across beakers. Incubations were set up identically to DOC incubations except that beakers were sealed airtight using low-density polyethylene film (Saran™) bound by rubber bands and were organized in replicates of 5 for each primary producer along with 5 controls containing filtered backreef seawater only. Initial DO readings were obtained from each beaker using a HACH LANGE HQ40 multiparameter instrument (DO: precision 0.01 mg l^−1^, accuracy±1%). After initial measurements beakers were sealed airtight and incubated in the dark at *in-situ* temperature for 45 min to determine dark respiration rates for each organism. For repeated DO readings, sensor tips were carefully threaded through the tightened lids to minimize gas exchange and two readings per replicate were measured over a 2–5 min period. Treatments were then exposed to 5 successively increasing PAR intensities (∼30, ∼60,∼120,∼300, and ∼600 µmol quanta m^−2^ s^−1^), for 45 min each. PAR was measured using the light sensor on a WALZ diving-PAM underwater fluorometer. All daylight incubations ended with the sample in full ambient light and final daylight readings of DO were taken just before sunset to determine the daily net oxygen production for each replicate. Incubations were then left for 12 h at *in situ* temperature in the dark and final measurements were taken the next morning to simulate a whole diurnal cycle.

### Microbial respiration, DOC consumption, abundance and activity

To determine if DOC released by different primary producers had an effect on the ambient backreef microbial populations and on water chemistry, we performed ∼48 h dark dilution culture incubations and measured changes in concentrations of DO, DOC, and bacterioplankton over time. The seawater remaining in each replicate beaker following the DOC release incubations detailed above (680 ml) was filtered through a pre-flushed (1 L low-organic deionized water; Barnstead Nanopure) 0.2 µm polyethersulfone filter (Pall SUPOR-200) and then inoculated (500 mL incubation water:200 mL inoculum) with freshly collected unfiltered backreef seawater (inoculum) to add a compositionally-representative ambient microbial community to the sample [32]. Sub-samples were taken from each treatment and initial oxygen concentration of each sub-sample was determined using the HACH LANGE DO optode sensor. Sub-samples were then kept in the dark at *in situ* temperature (26±0.5°C) in airtight ground-glass stoppered bottles (Wheaton BOD 120 ml). After 45–50 h, DO was measured again as described above. To calculate planktonic microbial oxygen consumption rates, the end oxygen concentration value was subtracted from start value and data were normalized by the duration of the incubation. These data provide estimates of microbial respiration over the time of the inoculation.

An additional parallel incubation was performed to determine the amount of DOC that was consumed by the microbial community from the different primary producer treatments and to measure changes in microbial abundance over time. The remaining inoculated incubation water from the light incubations was transferred into 250 mL acid-cleaned and sample-rinsed polycarbonate bottles (Nalgene). Initial water samples were taken to measure total organic carbon concentration (TOC; DOC plus bacterial carbon) and bacterial cell abundance. Incubation bottles were then kept at *in-situ* temperature in the dark over a time period of 45 to 50 h before sampling TOC again at the final timepoint. TOC **s**amples (40 ml) were collected in precombusted glass vials and stored at −20°C for up to four months until analysis via high temperature catalytic oxidation according to Carlson et al. [65]. Samples for bacterioplankton cell abundances (1–2 mL) in the incubations were collected roughly every 8 hours for the 45–50 h experiment, fixed in 0.5% paraformaldehyde and flash frozen at -80°C and stored for 2 months, and counted after 1X SYBR Green I (Invitrogen) staining using flow cytometry according to Nelson et al. [63].

### Data processing and derived variables

Rates of change in carbon concentration in both light incubations and dark dilution culture incubations and consumption of DO in dilution cultures were calculated by dividing the difference between start and end concentrations by the incubation duration (8.5 and 45–60 hours respectively). Rates of change for DO in the daytime primary producer incubations were calculated over 30 to 120 min of peak primary production rates at irradiance levels of 600 PAR µmol quanta m^−2^ s^−1^ (lux were converted to µmol quanta m^−2^ s^−1^ PAR according to the approximation established by Valiela [66]: 1 µmol quanta m^−2^ s^−1^ 400–700 nm = 51.2 lux) because it resembled the average daylight PAR availability and coincided with highest photosynthetic performance for all investigated organisms. Nighttime rates of change in DO were calculated over ∼12 h as described above. We calculated proportional release of photosynthate as the ratio of DOC:DO during daylight incubations; because DOC release rates during the whole daylight incubation period (8.5 hours) were compared to oxygen evolution rates only at peak photosynthetic yields, estimates of the proportional release of photosynthetically fixed organic carbon as DOC are likely to be conservative.

Bacterial cell yields were calculated as the increase in cells from start to end of the incubation, and bacterioplankton specific growth rates were calculated as the natural log change in abundance over the period of loglinear growth (roughly hours 8 to 30 of the dark incubations). Bacterioplankton abundance was converted to carbon units assuming 20 fg C cell^−1^ [67] and DOC in the dark dilution cultures was calculated by subtracting bacterial carbon from measured TOC. Bacterial growth efficiency (BGE) was calculated as the ratio of bacterial carbon production (rate of increase in bacterial carbon) to bacterial carbon demand (BCD; rate of removal of DOC).

Surface area-normalized rates of change in DOC and DO were calculated by subtracting changes in controls and dividing rates by the surface area of each benthic macroorganism incubated in each starting replicate beaker; surface area was chosen as common reference parameter because of its functional importance as an ecological interface with the surrounding environment and relevance for similarities in light capturing abilities [68]. For the samples of *Porites*, turf algae and CCA, the surface area was measured directly according to the advanced geometry method described by Naumann et al. [69]. Surface area of the remaining specimens were estimated by measuring their dry weight and then applying conversion factors established by Russo [70] for *Amansia* and *Turbinaria* and Haas and Wild [48] for *Halimeda*, respectively.

To estimate how different benthic assemblages could potentially contribute to DOC availability and subsequent microbial activity on coral reefs, a model was constructed using the DOC release and microbial growth rates measured in this study. A matrix was generated for four coarse functional groups of benthic organisms (coral, CCA, macroalgae, and turf algae) and for nine graduations of coverage with a combined total of 100%. The matrix generated 102 possible combinations of benthic coverage. Mean surface-normalized DOC release and bacteria yield measured in the incubation experiments were used to extrapolate concentrations of DOC and bacteria generated by different coral reef benthic assemblages, respectively.

### Statistical analysis

Statistics were performed using SAS within the software package JMP (v9; SAS institute 1989–2011). All statistical tests were conducted on log-transformed data to meet assumptions of normality unless noted otherwise; transformation improved normality on all metrics and after transformation median skewness was 0.14 (range −1.2 to 1.1) and median kurtosis was −0.06 (range −0.6 to 1.8) among 15 derived variables. Mean rate measurements (daylight DOC and DO release, dark DOC and DO removal and bacterial cell yield) in each treatment were tested for significant differences from zero using pairwise two-tailed t-tests among each replicate incubation vessel; data were untransformed in these tests to allow direct comparison with the null hypothesis of 0 change (zero change cannot be used as a null hypothesis with log-transformed data). We tested whether stocks of DOC, DO, or bacterioplankton differed significantly from ambient conditions and whether derived variables (rates and ratios) differed from control treatments using analysis of variance (ANOVA) followed by Dunnett's *post hoc* test with α = 0.05. We tested whether rates differed among the six treatments using Tukey's *post hoc* tests with. Five separate untreated control experiments were run daily (each in parallel with one or two treatment incubations); because controls did not differ significantly from each other in any of the measured rates or other derived variables (ANOVA, F_4,15_<3.5, p>0.05) for simplicity of presentation and analysis in all *post hoc* statistical comparisons we pooled controls.

### DOC analytical consistency and contaminant detection

Mean and median analytical coefficients of variation in DOC analyses were 1.3% [65]. We removed two sets of TOC samples from analysis due to contamination as follows. In one incubation experiment (17 Sept. 2011; treatments CCA and Turf) all dark dilution culture replicates exhibited a consistent increase in TOC averaging 5 µmol L^−1^ (pairwise two-tailed t-test p = 0.002) with no difference in magnitude of increase among treatments (ANOVA, F_2,13_ = 0.41, p = 0.67), indicating contamination of a single set of final timepoint sample collection bottles. Carbon parameters were not calculated from these incubations. We note that there was no evidence of contamination of the incubations themselves (both growth rates and oxygen removal rates were consistent relative to other treatments) and other parameters were analyzed accordingly. Additional evidence of TOC sample collection bottle contamination was found in four additional control samples, which were removed as outliers (concentrations>83 µmol L^−1^ and exceeding 95% of the 60 control TOC samples at a tolerance interval of α = 0.05).

## Results

### Experimental light and temperature conditions

Average backreef *in situ* daytime (09:00 to 17:00 h) PAR availability at ∼1 m water depth was 580±6 µmol quanta m^−2^ s^−1^ over the entire study period. Light loggers deployed in incubation vessels recorded an average PAR of 622±8 µmol quanta m^−2^ s^−1^ during daylight incubation hours. Average backreef *in situ* water temperature was 26.06°C with diurnal fluctuations of 2.51±0.40°C. Average water temperature in incubation chambers was 26.46°C with diurnal fluctuations of 3.21±0.51°C (Figure S1).

### Changes in DOC and DO in benthic producer incubations

Concentrations of DOC and DO in all light treatment primary producer incubations exhibited changes significantly different from zero over the course of the 8 hour incubations (p<0.01) and rates of release in all treatments were significantly greater than the controls (Dunnett's p<0.01; Figure S2). In the light control incubations DOC increased slightly, averaging 0.4 µmol L^−1^ h^−1^ (std. dev. 0.54; p = 0.02) but changes in DO were not significantly different from zero (p = 0.18). Starting concentrations of DOC in controls, turf, *Porites*, and CCA did not differ from ambient reef concentrations (68–73 µmol L^−1^, Dunnett's p>0.8) while starting DOC concentrations were elevated in all three macroalgal treatments (averaging 88 to 95 µmol L^−1^) as expected due to carryover contamination from the organisms and/or immediate DOC release associated with stress. Rates of oxygen consumption at night as a result of respiration were significantly different from zero in all treatments (pairwise p<0.02) and were greater than the controls (Dunnett's p<0.001).

### Surface area-normalized and control-corrected rates of DOC and DO release

**Figure 2:**
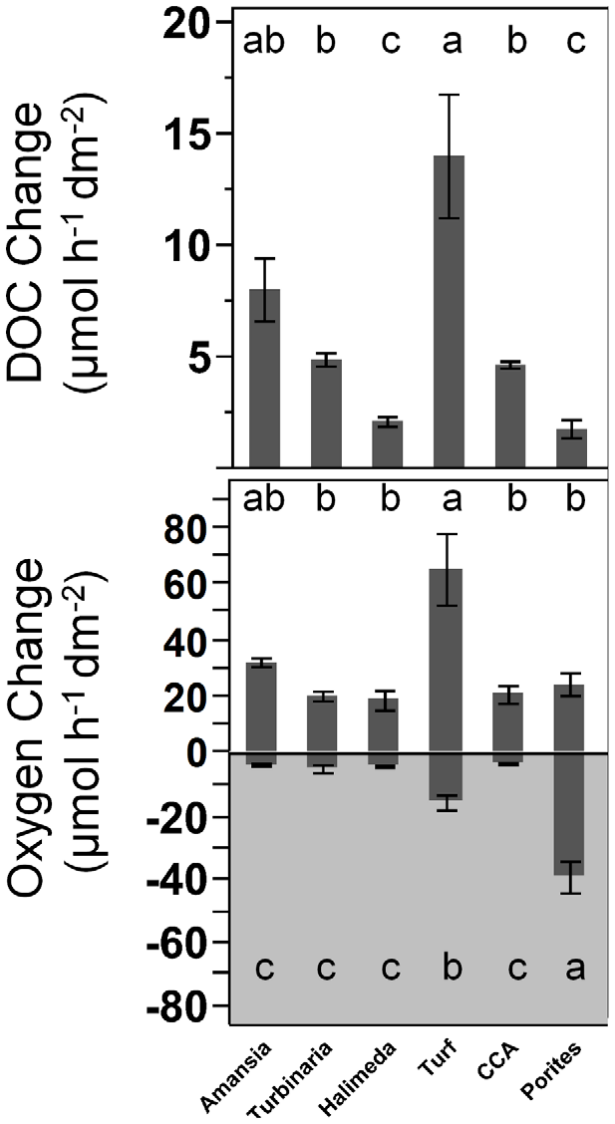
Rates of DOC production and DO production and consumption, normalized to surface area of benthic primary producers. Bars are means with standard error whiskers. Panels with grey background shading are dark incubations. A) Rates of DOC production during 8 h daylight incubation and B) oxygen production and consumption over a 24 hour light/dark cycle; treatments with the same letter are not significantly different at α = 0.05.

To allow for comparison between the different taxa, rates of DOC and DO production were corrected by subtracting mean control rates determined on each incubation date and normalizing individual replicate rates to surface area of the given organism (described above in methods). Surface-area normalized rates of DOC production ranged from 0.4 to 24.2 µmol h^−1^ dm^−2^, with the highest rates found in turf algae (14.0±2.8 µmol h^−1^ dm^−2^) followed by *Amansia* (8.0±1.4 µmol h^−1^ dm^−2^), while *Porites* and *Halimeda* had significantly lower DOC release rates (Figure 2A). Turf algae also demonstrated the highest oxygen production rates (Figure 2B) for all species investigated. Rates of daytime oxygen production were on average 2 to 10 times greater than nighttime consumption rates with the exception of *Porites,* which functioned heterotrophically by consuming more oxygen at night than was produced during the day (Figure 2B).

### Relationships between oxygen and carbon release

**Figure 3:**
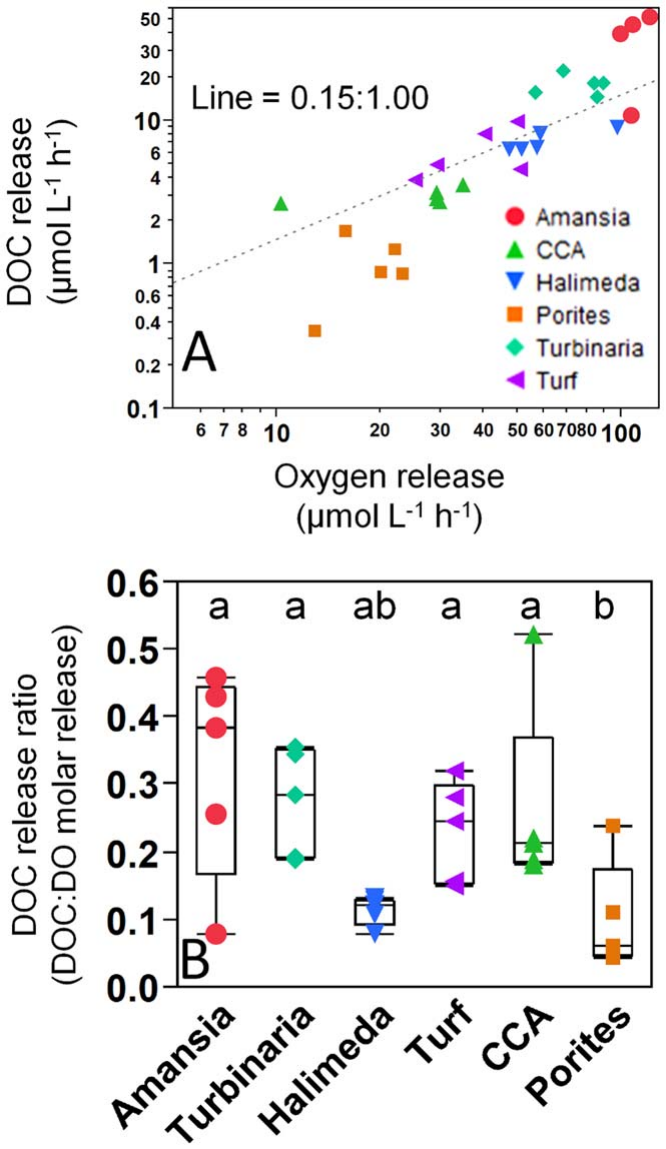
Relationships between oxygen and DOC release in Light incubations. Panel A relates absolute rates of oxygen and carbon production in light incubations, with line representing constant ratio of release for reference. Panel B shows variation in ratios of oxygen and DOC production among the six treatments; treatments with the same letter are not significantly different at α = 0.05. Quantile box plots in Panel B (whiskers extend to 95% quantiles) are overlaid with raw replicate data points from each treatment color/symbol coded according to the legend in panel (A).

Release rates of DOC were highly correlated with rates of photosynthetic oxygen release in the light incubations (r = 0.91, p<0.001, Figure 3A). Molar release ratios of DOC:DO ranged from 0.05 to 0.52 (averaged 0.21; Figure 3B), were greater than zero in all treatments (p<0.05) and differed significantly among treatments (ANOVA, F_5,30_ = 5.33, p = 0.002). Highest values were seen in the *Amansia* treatments (0.32) while *Porites* (0.11) was significantly lower than all other taxa. Using oxygen production as a proxy for photosynthesis, on average all benthic producers released at least 10% of their peak photosynthate as DOC into the surrounding waters (Figure 3B).

### Microbial consumption and respiration of exudates released by benthic producers

**Figure 4:**
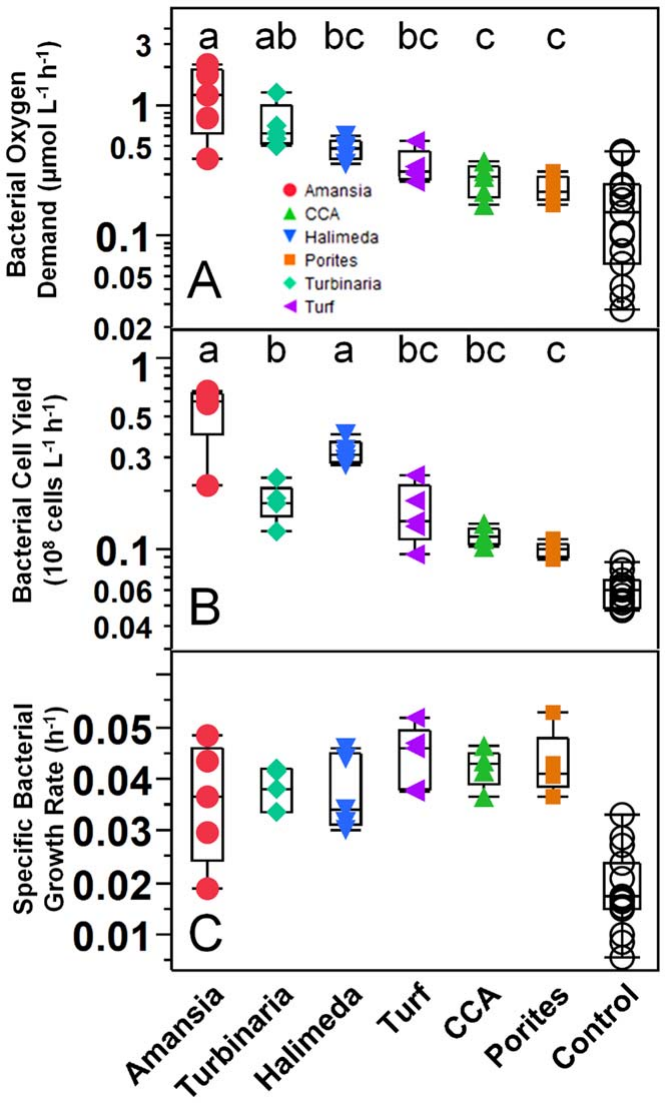
Quantiles of bacterioplankton growth and oxygen consumption in dilution culture incubations on different exudates. Panels show rates of bacterial oxygen consumption (A), bacterial cell yield (B), and bacterial specific growth rate (C). Quantile box plots (whiskers extend to 95% quantiles) are overlaid with raw replicate data points from each treatment color/symbol coded according to the legend in panel (A) and Fig. 3; treatments with the same letter are not significantly different at α = 0.05. Note that there were no significant differences among treatments in specific growth rate.

In dark dilution cultures, in which ambient bacterioplankton communities were grown on producer exudates, all measured treatments showed significant consumption of oxygen (Figure 4A) and DOC over time (pairwise two-tailed t-test p<0.05) with the exception of *Porites* exudates, which showed no significant change in DOC concentrations (pairwise two-tailed t-test p = 0.13; Table 1, note that DOC removal could not be assessed for turf algae and CCA due to sample bottle contamination). All treatments except CCA and *Porites* exhibited significantly higher rates of oxygen removal than controls (Dunnett's p<0.05). Oxygen declines averaged 0.55 µmol L^−1^ h^−1^ with average rates ranging from 0.32 µmol L^−1^ h^−1^ for the turf algae treatment to 2.1µmol L^−1^ h^−1^ for the *Amansia* treatment (Table 1, ANOVA, F_5,30_ = 12.40, p<0.001). In unamended controls rates of bacterial carbon demand (BCD, measured as the removal rate of DOC) and DO removal rates were significantly different from zero, averaging 0.07 and 0.18 µmol L^−1^ h^−1^, respectively (Table 1; std. dev. 0.04 and 0.14, respectively; p<0.001). Among the algal exudate treatments rates of BCD averaged 0.68 µmol L^−1^ h^−1^ with maximum rates exceeding 3 µmol L^−1^ h^−1^, and differed significantly among the four treatments analyzed (Table 1; ANOVA, F_3,20_ = 17.84, p<0.001); microbial communities grown on *Amansia* exudate exhibited the highest BCD amongst the four treatments measured.

**Table 1:** Starting concentrations and drawdown of TOC, Bacterial carbon demand (BCD), and bacterial growth efficiency (BGE) on DOM released by benthic producers.

	TOC Start (µmol L^−1^)				TOC Change (µmol L^−1^)					BCD (µmol DOC removed L−1 h^−1^)					BGE			
Exudate	Min	Med	Max	Tukey	Min	Med	Max	pairwise	Tukey	Min	Med	Max	pairwise	Tukey	Min	Med	Max	Tukey
*Amansia*	148.8	238.4	339.2	***A***	-18.4	-68.0	-121.4	***0.023***	***A***	0.50	1.80	3.15	***0.021***	***A***	0.03	0.06	0.09	***C***
*Turbinaria*	179.8	191.6	213.9	***A***	−9.3	−20.6	−26.3	***0.003***	***B***	0.21	0.44	0.56	***0.002***	***B***	0.05	0.07	0.13	BC
*Halimeda*	116.0	123.1	132.9	***B***	−5.1	−13.2	−15.5	***0.003***	***BC***	0.18	0.36	0.40	***0.001***	***B***	0.12	0.16	0.37	A
*Porites*	73.6	80.2	82.6	C	0.2	−1.4	−6.6	0.135	CD	0.01	0.05	0.16	0.080	C	*	*	*	*
Control	69.3	72.5	75.4	C	−0.9	−2.8	−5.0	***0.001***	D	0.03	0.07	0.13	***0.000***	C	0.06	0.14	0.32	AB

For each parameter minimum, median, and maximum values are reported (min, med, max) rather than means because of the variation in size of benthic organism in each incubation. Pairwise two-tailed t-test p-values are reported (pairwise) for rates to determine if changes from the start to the end of the incubation differed significantly from zero. Tukey tests are reported comparing means among treatments (different letters indicate significant difference in means at α = 0.05; letters in bold italic denote treatments significantly different from control treatments). *Cultures amended with *Porites* exudates showed no significant change in DOC over the course of the incubation and therefore BGE could not be estimated. Note also that, due to sample collection vial contamination DOC removal in cultures amended with CCA and Turf exudates are not shown.

### Bacterioplankton growth on DOC released by benthic producers

Bacterioplankton cell density increased significantly in all treatment and control incubations (Figure S3; pairwise two-tailed t-test p<0.001); this is expected in dilution culture and is primarily due to release of grazing pressure, allowing resolution of loglinear growth curves. Log-phase specific growth rates and final cell yields were significantly higher in all treatments relative to controls (Dunnett's p<0.001); mean specific growth rates in treatments (0.84 to 1.05 d^−1^) were more than double that of the controls (0.44 d^−1^) and mean cell yields in treatments (2 to 13×10^8^ cells L^−1^ d^−1^) were 1.7 to 9 times greater than the yield observed in the unamended control treatments of 1.4×10^8^ cells L^−1^ d^−1^ (Figures 4B, C). Ambient backreef bacterioplankton inoculum densities ranged from 2–5×10^8^ cells L^−1^ throughout the study period. All treatments had starting bacterial concentrations significantly less than ambient after dilution (Dunnett's p<0.05) with controls reaching ambient densities over the course of the 2-day incubations (Figure S3). There were significant differences in bacterioplankton cell yields among the six primary producer amended DOC treatments (Figure 4B; ANOVA, F_5,30_ = 25.44, p<0.001). *Amansia* and *Halimeda* exhibited highest bacterioplankton yields with *Porites* and CCA having significantly lower yields. However, we found no significant differences in log-phase specific growth rates among the six benthic producer treatments (Figure 4C; ANOVA, F_5,30_ = 1.318, p = 0.29). Bacterial growth efficiencies (the ratio of bacterioplankton carbon to BCD) ranged from 0.03 to 0.37 with a median of 0.10 and differed significantly among the four treatments analyzed (Table 1; ANOVA, F_3,24_ = 7.04, p = 0.002), with *Amansia* significantly lower than the control (*Porites* BGE was uninterpretable because TOC drawdown was not significantly different from zero). Bacterial cell carbon yield (but not specific growth rate) was a strong predictor of both BCD and oxygen consumption (r^2^ = 0.66 and 0.68, respectively; p<0.0001) across experiments, but there were no significant relationships within any individual treatment.

**Figure 5:**
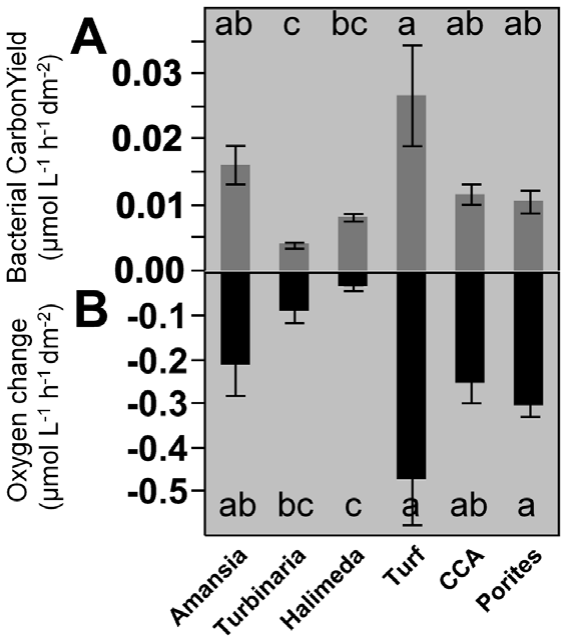
Rates of A.) bacterial carbon yield and B.) DO consumption on exudates over ∼48 hour dilution culture incubations normalized to surface area of previously incubated primary producers. Bars are means with standard error whiskers; treatments with the same letter are not significantly different at α = 0.05.

To account for the different algal sizes in each replicate and, subsequently, the different concentrations of exudates added to the bacterial cultures, bacterial carbon yield and oxygen removal were calculated when normalized to the surface area of the algal samples and after subtraction of control treatment drawdown rates. These surface area-normalized rates of bacterial carbon yield (Figure 5A) and concomitant removal of oxygen (Figure 5B) differed significantly among the six benthic producer treatments (ANOVA, F_5,29_>9, p<0.001). In general differences among treatments mirrored those of rates of DOC release and primary production (Figures 2 & 5), with the greatest surface area-normalized rates of bacterial yield and concomitant oxygen drawdown were found on exudates from turf algae and the lowest on *Turbinaria* and *Halimeda* exudates. However, the elevated growth efficiency of bacterioplankton on exudates from *Halimeda* (Table 1) and *Porites* (based on cell growth exceeding controls when carbon and oxygen drawdown did not differ from controls; Figure 4, Table 1) reiterate that exudates from these two taxa produced relatively large bacterial carbon yields (Figure 5A) relative to their moderate carbon and oxygen production (Figures 2A, B) and removal rates (Figure 5B).

## Discussion

This study was conducted to examine the influence of common coral reef associated benthic primary producers on ambient DOC and DO concentrations and the influence of the exuded DOC on microbial abundance and activity in their surroundings. Here we show that all six benthic reef taxa investigated had a significant, but varying influence on DOC, DO and the ambient planktonic microbial community. There were, however clear differences between benthic producer taxa in many of the measured rates, emphasizing the variable influence of primary producers on water chemistry and microbial processes. In complex ecosystems such as coral reefs [71], which are continuously subjected to natural and / or anthropogenic disturbances [72]–[76], these primary producer-induced intrinsic processes may play important roles in determining how coral reef ecosystems respond to disturbances. Organic material released by corals primarily as mucus [22], [77] has been shown to support nutrient cycling in the reef by functioning as particle trap, thereby introducing essential nutrients into the otherwise typically nutrient limited coral reef ecosystem [8]. In contrast, organic matter released by algae is generally dissolved [18], [46], [47] and has been hypothesized to affect coral health and potentially create a positive feedback facilitating the formation of ecosystem phase-shifts from coral- to algal dominance [45]. The effects of other primary producer induced alterations on biogeochemical parameters (e.g. oxygen availability and microbial abundance) have rarely been studied [54], [56], [78], [79], [80]. The data presented here provide the first species-specific, quantitative data on biogeochemical alterations and microbial growth facilitated by dominant primary producers in a typical shallow, backreef environment in the tropical Pacific.

### Influences of benthic primary producers on DOC concentrations

As has been shown with many primary producers across global ecosystems, the benthic algae examined here all released a significant proportion of their daily fixed carbon as DOC into their surroundings during daylight hours (Figure 2A). Conservative estimates suggest that at least 10% of the photosynthetically fixed organic carbon was exuded by turf and macroalgae (Figure 3B). These findings are in agreement with the limited data from previous studies on primary producer organic matter release conducted with comparable methodology in other coral reef ecosystems (average release rates of 4.3±0.7% [18] to 50.6±2.6% [81]) around the world. Turf algae from the Northern Red Sea have been shown to release more DOC than several other species of benthic primary producers (i.e. various macroalgae and corals), with release rates of 5.3–55.3 µmol h^−1^ dm^−2^ [46] bracketing the release rates estimated in the present study (14.0±2.8 µmol h^−1^ dm^−2^). Similarly, incubation experiments from the Mexican Caribbean found that red algae had DOC release rates of 10.3±8.5 µmol h^−1^ dm^−2^ [47] which were on average higher but in the same range as release rates measured here for the red alga *Amansia* (8.0±1.4 µmol h^−1^ dm^−2^). Greater DOC release rates in the Mexican Caribbean may be a result of overall higher water temperatures (30.4°C compared to 26.1°C) enhancing the metabolic performance of the species [82], [83]. Higher metabolic rates are common in algae with simple growth forms or high surface area to volume ratios, such as sheet-like or blade forming taxa (*Ulva, Amansia*) and especially filamentous algae (turfs) in comparison to thick leathery (*Turbinaria*), calcareous (*Halimeda*) and crustose (CCA) growth forms [46], [84]. These metabolic trends among algal types were supported by our data on primary production and DOC release rates (Figure 2). Increased metabolic rates found for algae with more simple growth forms are likely due to greater surface area to volume ratio, and thus greater area exposed to resources such as light and nutrients which allows for a faster exchange of metabolic products with the environment [85], [86], [87].

The coral examined here, *Porites lobata* exuded the smallest amount of DOC (Figure 2). Previous studies on DOC release rates of hermatypic corals have shown that coral DOC fluxes may vary considerably. Some studies reported significant net DOC release by corals [20], [21], [22], with release rates ranging from ∼1.2 µmol h^−1^ dm^−2^ surface area [88] to ∼3.7 µmol h^−1^ dm^−2^ [89]. In contrast, experiments on coral DOC release in the Red Sea [77] and the Mexican Caribbean [47] showed highly variable but on average insignificant contribution of corals to the ambient DOC pool. The observed differences are possibly due to the varying degrees of autotrophy versus heterotrophy in corals [54]. Besides using photosynthetic products from zooxanthellae [90], the coral host may also take up DOC from the surrounding water column [91], [92]. Here *Porites* had comparably low oxygen release rates, while exhibiting high respiratory oxygen demand during dark incubations. Thus, *Porites* had a net negative diurnal oxygen balance suggesting that these coral holobionts were net heterotrophic (Figure 2B). The degree of autotrophy versus heterotrophy of coral holobionts is clearly variable [93] and depends largely on resource availability (light, zooplankton, dissolved compounds, etc). These patterns suggest that direct DOC uptake by the corals or coral-associated microorganisms may have compensated for release rates resulting in net neutral DOC fluxes during daylight incubations.

### Influences of benthic primary producers on oxygen concentrations

All of the primary producers examined here significantly increased oxygen concentration during the day as a result of photosynthesis and consumed oxygen due to respiration at night (Figure 2B). Similar to patterns observed with DOC release, algae with filamentous and sheet like growth forms (turf algae, *Amansia*) had the highest oxygen release rates and photosynthetic performance. These findings further support the idea that algae with high metabolic rates are also likely to be more “leaky” and release a greater proportion of their daily photosynthetically fixed carbon into the surrounding seawater.

The high metabolic oxygen demand of the coral and the turf algae consortium at night highlights the role of oxygen availability [45], [94] in the outcome of competitive interactions particularly of these two organism types. Respiration of the coral holobiont (including the coral host, the symbiotic algae, and the coral-associated microbial community) and the adjoining turf algae, accompanied by increased planktonic microbial oxygen demand, facilitated by DOC release from coral and algae may lead to extremely low DO concentrations in the immediate surroundings of these organisms [57], [58], [95]. Hypoxic stress noticeably increases the vulnerability of corals to invasive organisms [96] and may lead to release of the symbiotic zooxanthellae (bleaching) induced by an increased production of lactic acids through anaerobic respiration in hypoxic conditions, which causes an acidic environment unfit for the symbiotic algae [96], [97]. Further, superoxide dismutase activity declines [98], and later re-oxygenation of the hypoxic tissue could lead to severe cellular damage [99]. Benthic macroalgae on the contrary have been shown to temporarily tolerate conditions of very low (≈30 µmol L^−1^) oxygen availability [100], thus giving algae a competitive advantage in interaction zones experiencing localized hypoxia.

### Variations in DOC quality and microbial response among algal exudates

Bacterial specific growth rates on substrates released from benthic producers were nearly double those of ambient bacterioplankton communities, suggesting that all investigated exudates contain DOC which is more labile than the ambient pool (Figure 4C). Benthic producers with the most rapid DOC release rates engendered the most rapid and least efficient microbial respiration: exudates from producers with the highest surface-area normalized rates of DOC release (Figure 2A) yielded the highest surface area-normalized rates of DO removal (Figure 5B; correlation p = 0.006, r = 0.50) and the lowest bacterial growth efficiences (Table 1; correlation p = 0.002, r = -0.66). Notably, bacterioplankton growth rates and yields were uncorrelated with DOC release rates (p>0.1), emphasizing that these patterns in efficiency were not solely driven by the absolute quantity of cells produced, but also by the utilization of the DOM exuded. Among the algae, *Turbinaria* exhibited relatively low bacterial cell yields (Figure 4B) and specific DOC removal rates, despite having relatively high rates of DOC release (Figure 2A), translating into significantly reduced bacterial growth efficiency. In contrast, *Halimeda* exhibited one of the lowest rates of DOC production (Figure 2A) and much lower DOC release ratios than the other organisms, but the produced DOC exhibited relatively high bacterial yields (Figure 4B) translating into significantly higher growth efficiencies than the other treatments (on par with growth efficiency on ambient DOC; Table 1). These patterns in the efficiency of production and removal of DOC together suggest that *Turbinaria* has relatively low-quality DOC (i.e. low growth efficiency) and *Halimeda* relatively high-quality DOC (i.e. high growth efficiency) compared with other benthic producers examined here. We also note that *Amansia* appeared to produce large amounts of highly labile DOC with low bacterial growth efficiencies (Table 1), suggesting selection for a highly inefficient community growing on the rapidly exuded compounds.

### Coral exudate DOC quality and microbial response

DOC dynamics in incubations with the hermatypic coral *Porites* showed a significant departure from the patterns observed with the different algae. First, the relatively high rates of respiration and low rates of DOC release suggest that the corals or their associated microbial consortia rapidly consume a portion of their exuded DOC (Figure 3). Second, while rates of drawdown of DOC and oxygen in dilution cultures amended with *Porites* exudates did not differ significantly from controls (Table 1, Figure 4a), bacterial growth rates and yields and oxygen consumption rates of *Porites* treatments were significantly greater than controls and were similar to results from CCA exudates (Figure 4b–c). Previous studies have suggested that DOC released by hermatypic corals has no, or even decreasing effects on subsequent microbial oxygen consumption rates [47]. They therefore assigned increases in subsequent planktonic microbial oxygen consumption rates by the elevated concentrations of particulate organic carbon [47] released by corals as mucus [27]. The present study strictly excluded the particulate fraction of organic carbon by filtering the incubation water. Because cells showed enhanced growth on *Porites* exudates without enhanced DOC or DO removal, our data suggest that coral exudates may support a different microbial community metabolism using an alternate electron donor to fuel their metabolic processes than organic carbon.

### Ecological implications

Here we show that benthic coral reef primary producers can alter the biogeochemical cycles in their communities not only directly through physiological processes but indirectly via DOC-mediated interactions with bacterioplankton. The rapid degradation of algal-released DOC by microbes and the concomitant drawdown in DO highlights the significance of these interactions and the potential role that microbes play in coral-algae interactions [43], [44], [45]. Previous studies have hypothesized organism-specific differences in ecological properties (e.g. size, C/N ratio, lability) of primary producer exudates [31]. Our data show that the photosynthate released by some algal species may not only lead to elevated microbial activity, with potential implications on oxygen availability, but might also support a substantially different ambient microbial community compared to that of hermatypic corals.

**Figure 6:**
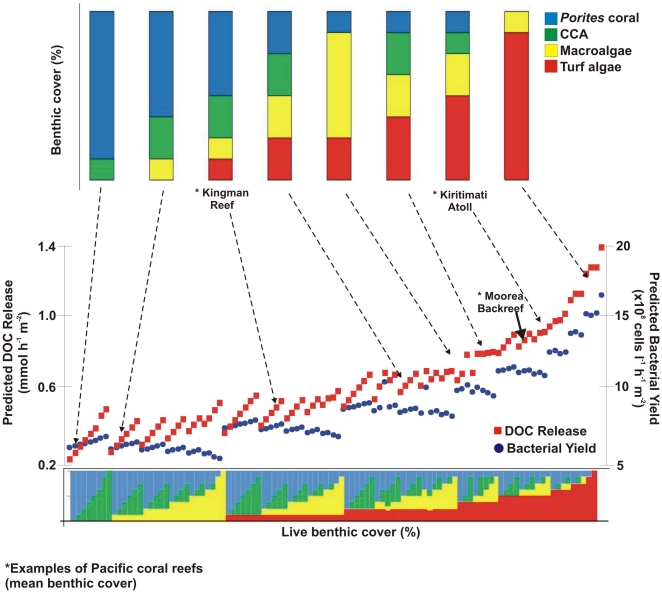
The predicted DOC release (red squares) and bacterial yield (blue circles) for varying coral reef benthic assemblages. The six benthic species characterized in this study were separated into four functional groups: turf algae, macroalgae (*Amansia rhodantha* and *Turbinaria ornata*), calcifying algae (*Halimeda opuntia* and *Hydrolithon reinboldii* (CCA)), and hermatypic coral (*Porites lobata*). Different combinations of percent cover for the four benthic functional groups were calculated (N = 102), the bar charts represent eight examples of the possible benthic assemblages. The measurements for benthic cover from two of the Line Islands (Kingman and Kiritimati) and Moorea were taken from Sandin et al, 2008 and the LTER database (knb-lter-mcr.8, http://mcr.lternet.edu/data), respectively.

The differences in microbial consumption rates of DOC and oxygen from exudates of corals and different reef algae further highlights the implications of shifts in the composition of benthic assemblages on coral reefs. For instance, the measurements of DOC release by turf algae and its influence on microbial activity were notably higher compared to other taxa. Experimental rate measurements from this study were used to extrapolate the average DOC release rates and bacterial yield of four coarse functional groups of photosynthetic benthic organisms for a given area of reef benthos. Figure 6 illustrates a model that predicts the DOC release rates and bacterial cell yield for different hypothetical benthic assemblages on coral reefs. Based on this model, reefs dominated by turf- and macroalgae will support 2–3 x the number of microbes than coral and CCA dominated reefs. These coarse estimates based on only a handful of taxa clearly represent an oversimplification of what likely occurs in a highly diverse natural reef environment. Caveats of using bacterial yield measurements from incubation experiments to extrapolate in situ reef conditions include overestimation of bacterial growth because of the diluted cultures. Nevertheless, previous research has demonstrated that reefs with higher abundances of fleshy algae maintain higher standing stocks of bacteria in the water column. Degraded coral reefs that are dominated by algae, such as the leeward reefs on Kiritimati atoll have been shown to support microbial abundances an order of magnitude higher than the coral-dominated reefs on Kingman [101], [102]. Turf algae is a well-known player in the succession of coral-to-fleshy algae-dominated reefs [103] and these results further support previous studies which suggest that microbial activity may contribute to this transition.

### Further research and conclusions

This study illustrates the interactions and dynamics associated with benthic primary producer physiology and reef microbiology. Further research is needed to examine how corals may be affected by different algal exudates and which roles these direct (primary producer respiration), or microbial mediated indirect effects (elevation of microbial oxygen consumption through exudates), play in coral-algal interaction events. Though it has been demonstrated that numerous mechanisms and competitive strategies exist (e.g. allelopathy, abrasion, and disease transmission) in the fierce battle for space on the coral reef benthos, more data is needed to elucidate the roles that microbes play in these interactions. The influence of the reef benthos on the surrounding ecosystem are also going to be highly dependent on physical oceanography and the residence time of overlying water bodies. Therefore these parameters should be included in studies which model carbon cycling on reefs. Finally, these data suggest that bacterioplankton dynamics are tightly linked to benthic community composition identifying that shifts in the composition of the benthos may have large effects on trophic structure and ecosystem functioning of coral reefs.

## Supporting Information

Figure S1 **Light availability and temperature regime** ***in situ*** **at the Moorea backreef location and in incubation beakers during daylight incubations.**
(TIF)

Figure S2 **Pairwise changes in concentrations of DOC and oxygen through time in the Light and Dark phases of incubation for each treatment.** Each line represents an individual replicate incubation vessel.
(TIF)

Figure S3 **Individual replicate bacterial growth curves for each benthic producer treatment and controls in the dark dilution cultures.** Ambient backreef bacterioplankton density ranges over the course of the experiments are highlighted with a gray band.
(TIF)
