# Supplementary figures and images for: Effects of Coral Reef Benthic Primary Producers on Dissolved Organic Carbon and Microbial Activity
Source: PLoS One. 2011 Nov 18;6(11):e27973. doi: 10.1371/journal.pone.0027973 (PMC3220721; doi:10.1371/journal.pone.0027973)

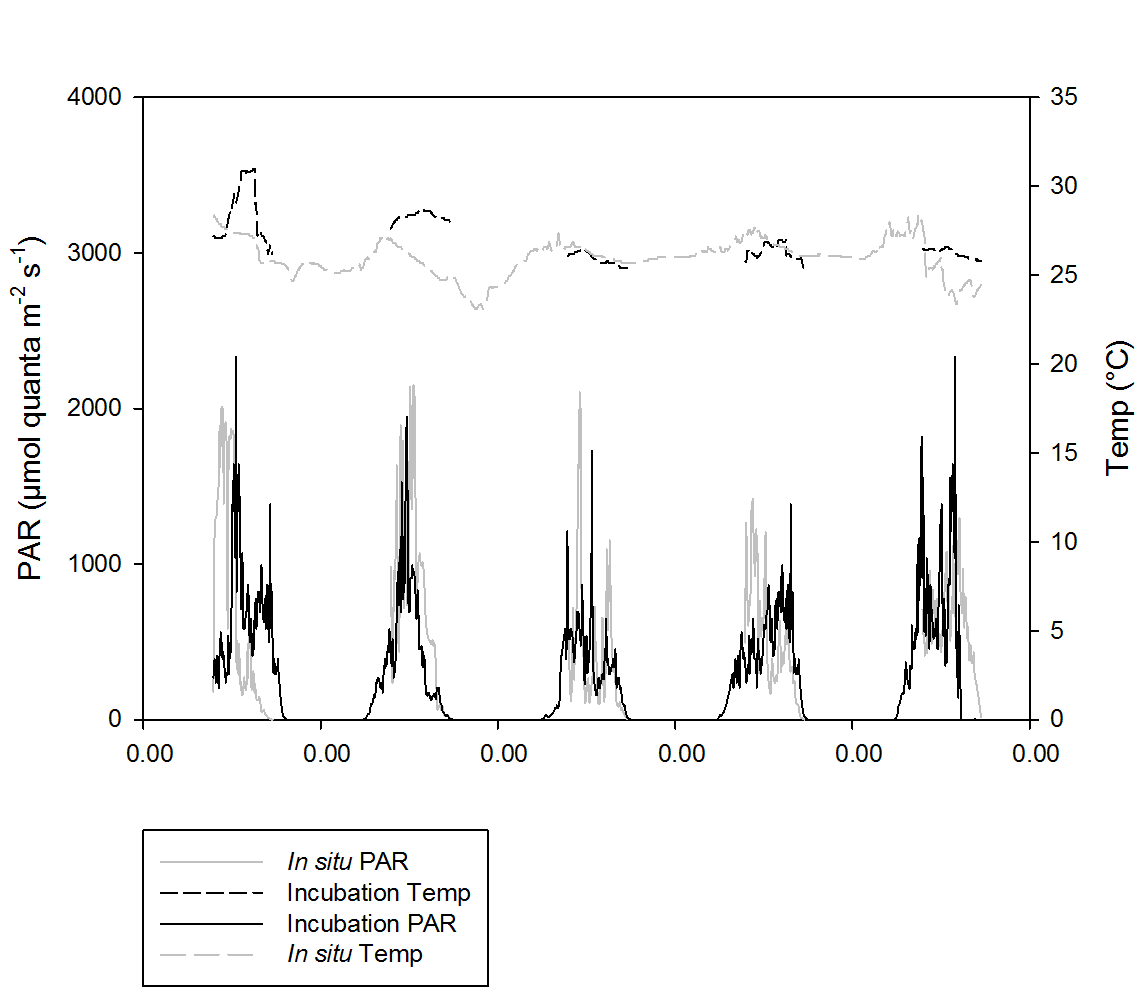

Supplement: Figure S1 — Light availability and temperature regime in situ at the Moorea backreef location and in incubation beakers during daylight incubations. (TIF) [file pone.0027973.s001.tif]
